# Dietary lipids, gut microbiota and lipid metabolism

**DOI:** 10.1007/s11154-019-09512-0

**Published:** 2019-11-09

**Authors:** Marc Schoeler, Robert Caesar

**Affiliations:** The Wallenberg Laboratory, Department of Molecular and Clinical Medicine, University of Gothenburg, 41345 Gothenburg, Sweden

**Keywords:** Diet, Dietary lipid, Lipid, Fatty acid, Gut microbiota, Microbiome, Dysbiosis, Dyslipidemia, Lipid metabolism, NAFLD, Non-alcoholic liver disease, TMA, TMAO, Trimethylamine N-oxide, Inflammation, Lipopolysaccharides, LPS, Gut permeability, FXR, TGR5, Bile acid, Atherosclerosis

## Abstract

The gut microbiota is a central regulator of host metabolism. The composition and function of the gut microbiota is dynamic and affected by diet properties such as the amount and composition of lipids. Hence, dietary lipids may influence host physiology through interaction with the gut microbiota. Lipids affect the gut microbiota both as substrates for bacterial metabolic processes, and by inhibiting bacterial growth by toxic influence. The gut microbiota has been shown to affect lipid metabolism and lipid levels in blood and tissues, both in mice and humans. Furthermore, diseases linked to dyslipidemia, such as non-alcoholic liver disease and atherosclerosis, are associated with changes in gut microbiota profile. The influence of the gut microbiota on host lipid metabolism may be mediated through metabolites produced by the gut microbiota such as short-chain fatty acids, secondary bile acids and trimethylamine and by pro-inflammatory bacterially derived factors such as lipopolysaccharide. Here we will review the association between gut microbiota, dietary lipids and lipid metabolism

## Introduction

The gut microbiota regulates many metabolic processes in the host including energy homeostasis, glucose metabolism and lipid metabolism [1]. Microbial imbalance, sometimes termed dysbiosis, is associated with metabolic perturbations, and several studies have demonstrated a causal relationship between microbial function and metabolic perturbations. Therapies targeted against the gut microbiota have been shown to improve the metabolic function in humans [2, 3] and transplantation of the fecal microbiota from patients with obesity, steatosis or type 2 diabetes can partly reproduce the donor’s metabolic phenotype in mouse recipients [4–7].

Lipid metabolism includes the biosynthesis and degradation of lipids such as fatty acids, triglycerides and cholesterol. Specialized lipoproteins facilitate the transport of lipids from the gut to the liver (the site of most lipid transformations) and between the liver and peripheral tissues. Obesity is linked to dysregulation of lipid metabolism, which may result in abnormal levels of blood lipids, ectopic lipid deposition and associated metabolic diseases such as non-alcoholic liver disease (NAFLD) [8] and atherosclerosis [9]. Lipid metabolism is mainly regulated by nutrients such as sugars and fatty acids. However, several reports have shown that lipid levels are associated with the gut microbiota composition, and mechanistic links between lipid metabolism and microbial metabolites have been described in mouse models.

The gut microbiota has the capacity to perform many processes that cannot be carried out by the host. These processes can give rise to microbially produced or modulated metabolites that function as metabolic substrates and signaling molecules in the host, with major implications for host metabolism and health. Dietary composition is central to the metabolic output of the gut microbiota because: (1) the gut microbiota processes dietary nutrients into metabolites and (2) the diet affects the gut microbiota composition and thereby its metabolic potential and impact on the host. In particular, the importance of dietary fibers for gut microbiota composition and function has been extensively studied. In addition, several studies have reported an important role for dietary lipids.

In this review, we will discuss interactions between lipids, the gut microbiota and the host. We will describe the current knowledge on how dietary lipids affect the gut microbiota, how interactions between dietary lipids and the gut microbiota influence host physiology and health, and how the gut microbiota affects host lipid metabolism.

## Interaction between dietary lipids and the gut microbiota

**Fig. 1:**
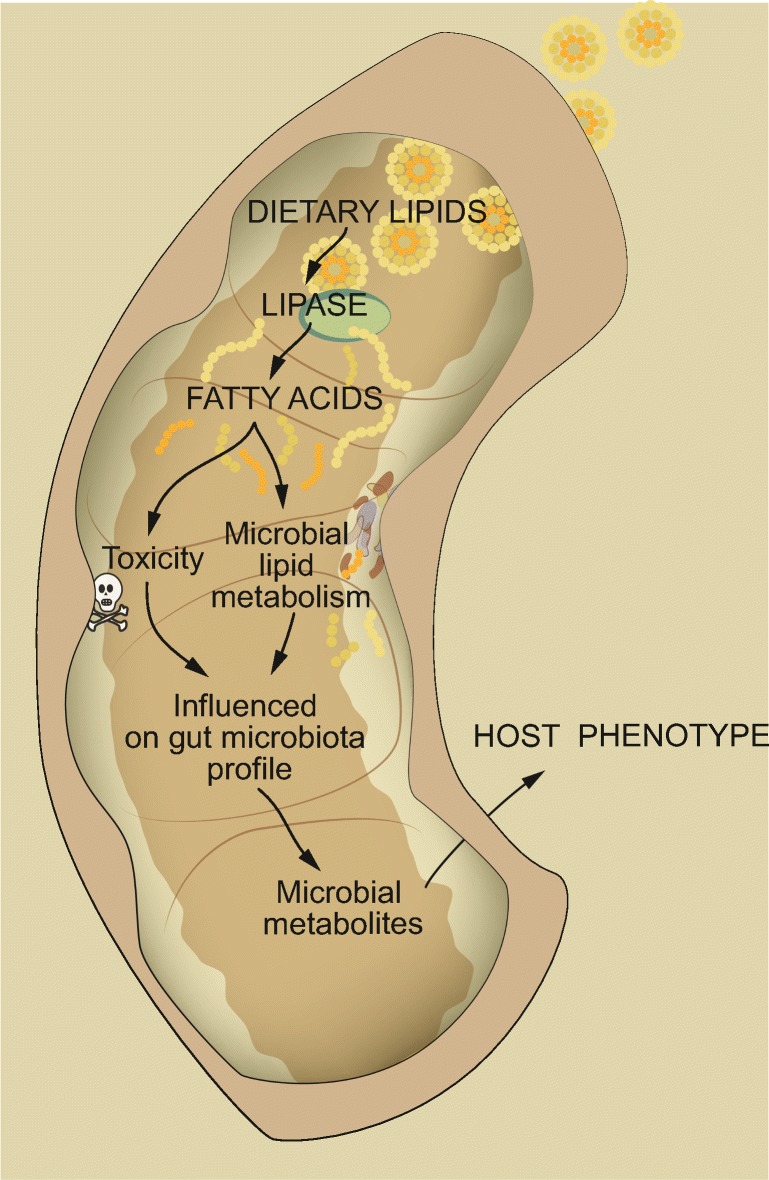
**Interaction between dietary lipids and the gut microbiota influences host physiology.** Free fatty acids are produced from lipid precursors by the action of lipases. Fatty acids may have antibacterial activity or may be utilized as metabolic substrates by gut bacteria, thereby affecting gut microbiota profile and production of microbial metabolites. This may influence host physiology and health

The gut microbiota has been shown to differ between mice fed diets that are high or low in fat and between diets that contain equal amounts of fat but from different sources [10–13] (Fig. 1). A comparison of mice on a variety of diets (low-fat diet and diets containing high levels of saturated fat, *n-6* PUFA or *n-3* PUFA) showed that diets with saturated fat or *n-6* PUFA induced weight gain, but only saturated fat increased insulin resistance, colonic permeability, and mesenteric fat inflammation [12]. The gut microbiota composition of mice fed a low-fat diet and a *n-3* PUFA diet differed from the other groups, whereas the gut microbiota of mice fed a saturated fat diet and a *n*-6 PUFA diet were similar. In another study where mice fed a high-fat diet containing lard, rich in saturated fat, were compared to mice fed a isocaloric high-fat diet containing fish oil, rich in *n-3* PUFA, it was found that phylogenetic diversity and abundance of the beneficial bacteria *Akkermansia muciniphila*, Lactobacillus and Bifidobacterium were lower in mice on a lard diet [10]. The lard diet also reduced insulin sensitivity and increased inflammation in white adipose tissue (WAT) through activation of Toll-like receptor 4 (TLR4) signaling. Transplantation of cecum microbiota into germ-free (GF) mice showed that the diet-induced differences in host phenotype were partly caused by the gut microbiota.

Not only fat sources with major differences in lipid composition, such as lard and fish oil, but also fat sources that are more similar, may give rise to different gut microbiota composition and function. Devkota et al showed that a diet with milk fat, but not diets with lard or safflower oil, increased expansion of *Bilophila wadsworthia* in mice [13]. Milk fat promoted taurine-conjugation of bile acids, which increases the availability of sulfur used by *B. wadsworthia*. The increased levels of *B. wadsworthia* were associated with a pro-inflammatory immune response and increased incidence of colitis in genetically susceptible mice. In another study that combined lard or palm oil with dietary bile acids, lard enhanced fat mass accumulation, impaired glucose tolerance, and elevated levels of hepatic triglycerides in conventionally raised (CONV-R) but not in GF mice when compared to palm oil [11]. The lard diet also promoted a shift in gut microbiota composition and functions, including changes with potential impact on lipid and amino acid metabolism.

The mechanisms by which dietary fatty acids affect gut microbiota are not well defined. Although most of the fatty acids consumed are absorbed in the small intestine, a minority will pass through the gastrointestinal tract and may therefore directly modulate colonic microbiota composition. Fatty acids have a broad spectrum of antibacterial activity including lysis and solubilization of bacterial cell membranes [14, 15] and inhibition of ATP production [16]. The antibacterial action of fatty acids is affected by carbon chain length, saturation and double bond position [17]. However, the impact of fatty acids on the gut microbiota is not limited to antibiotic action. Although gut anaerobes cannot produce energy by beta-oxidation, bacteria can metabolize fatty acids through other pathways. For example, in a mouse model of alcoholic liver disease, ethanol was found to inhibit biosynthesis of saturated fatty acids by the intestinal microflora. Dietary supplementation with saturated long-chain fatty acids, which were metabolized by and promoted growth of Lactobacillus, reversed alcohol-induced dysbiosis, stabilized the intestinal gut barrier, and reduced liver injury [18].

Intestinal bacteria can also react with fatty acid double bonds to produce metabolites that cannot be synthesized by mammalian hosts. Bacterial processing of linoleic acid, for example, has been shown to produce metabolites that may influence host physiology and health. Conjugated linoleic acid (CLA) can be produced by several gut bacteria including Lactobacillus, Butyrivibrio, and Megasphaera [19, 20]. Different CLA isomers have been demonstrated to have different, and sometimes opposite, effects on the host: c9,t11-CLA improves insulin sensitivity and decreases atherosclerosis by activation of proliferator-activated receptor γ (PPARγ), t10,c12-CLA worsens insulin sensitivity and atherosclerosis by inhibiting expression of PPARγ and LXRα [21–23], and t9,t11-CLA reduces atherosclerosis by activation of LXRα [24]. Different bacteria produce different ratios of CLA isomers [25, 26] and promotion of bacteria that produce high levels of beneficial CLAs could therefore potentially be used to promote a healthy metabolic phenotype.

Bacterial production of CLAs is a multistep process involving several metabolic intermediates. These metabolites include several hydroxy fatty acids that affect processes related to host health. 10-hydroxy-cis-12-octadecenoic acid (HYA) enhances intestinal barrier function and suppresses the development of colitis in mice in a free fatty acids 1 (FFR1/GPR40)-dependent manner [27]. Another hydroxylated CLA intermediate - 10-oxo-cis-12-octadecenoic acid (KetoA) - increases adiponectin production and glucose uptake in a PPARγ-dependent manner, and contributes to the prevention of obesity-related metabolic perturbations [28].

## Influence of the gut microbiota on host lipid metabolism and pathophysiology

Studies in gnotobiotic mice have shown that the gut microbiota affects host lipid metabolism. Importantly, GF mice are protected against diet-induced obesity through a combination of several mechanisms including increased fatty acid oxidation and decreased deposition of triglycerides in adipocytes compared to CONV-R mice [29]. Furthermore, lipidomics analysis of GF and CONV-R mice fed a regular chow diet showed that the gut microbiota affects lipid composition in host tissues and serum and increases clearance of triglycerides from the circulation [30]. In contrast, circulating triglycerides, HDL, and total cholesterol levels are increased by the gut microbiota in mice on a high-fat diet [31]. Comparisons between CONV-R and GF mice have also shown that the gut microbiota induces hepatic production of monounsaturated fatty acids and elongation of PUFA, and that acetate produced by the gut microbiota is used as precursor in hepatic fatty acid synthesis [32].

The gut microbiota affects host lipid metabolism and lipid composition through interaction with the diet. In a recent study by Just et al, CONV-R and GF mice were fed palm oil or lard diet (both rich in saturated lipids) supplemented with bile acids [11]. They found that the gut microbiota increased hepatic triglycerides and cholesteryl esters levels only in mice fed lard, and that colonization status had a major impact on hepatic lipids. In another study where CONV-R and GF mice were fed lard or fish oil diet it was also found that the microbiota downregulates cholesterol biosynthesis and increased hepatic levels of cholesterol specifically in mice fed lard [33]. However, in this study the relative contribution of the gut microbiota to the total variation in the hepatic lipid dataset was small and no serum lipids differed significantly between CONV-R and GF mice. This discrepancy between the two studies could possibly be attributed to the relative similarity between palm oil and lard compared to fish oil and lard.

Studies in mice treated with probiotics provide further evidence for a role of the gut microbiota in regulation of host lipid homeostasis. In mice fed a high-fat high-cholesterol diet, *Lactobacillus curvatus* alone or together with *Lactobacillus plantarum* reduced cholesterol in plasma and liver and the two strains had a synergistic effect on hepatic triglycerides [34]. Similarly, in obese rats fed a high-fat diet, Bifidobacterium spp. decreased levels of circulating triglycerides and LDL and increased levels of HDL [35].

Overall, studies in mouse models show that the gut microbiota, in concert with the diet, regulates host lipid metabolism and lipid levels in serum and tissues.

The fecal microbiota has also been linked to lipid metabolism in humans. Taxonomy and functional profiles of the bacteria differ between obese and lean subjects, but results from different studies are inconsistent, in part because of the complex nature of obesity but also because different methods have been used to analyze the microbiota [36]. A number of studies have investigated the association between the gut microbiota and dyslipidemia. When profiling metagenomics data from obese individuals, Cortillard et al found that reduced total microbial gene richness was associated with increased total serum cholesterol and serum triglycerides in obese patients. An energy-restricted diet intervention increased microbial gene richness and reduced serum lipids [37]. Similarly, Le Chantelier et al showed that triglycerides were higher and HDL levels were lower in individuals with low microbial gene counts than in those with high microbial gene counts [38]. Furthermore, by performing cross-validation analysis on taxonomic data of fecal microbiota, biometrics and metabolic measurements from a general population cohort study in the Netherlands, Fu et al could attribute 6% of variance of serum triglycerides and 4% in HDL to the gut microbiota composition [39].

Changes in fecal microbiota composition are also present in individuals with pathophysiological conditions associated with dyslipidemia and ectopic fat deposition such as atherosclerosis and fatty liver. By analyzing the microbial composition of atherosclerotic plaques, fecal samples and the oral cavity in patients with symptomatic carotid artery stenosis [40], Koren et al observed shared operational taxonomic units (OTUs) between all three sites, consistent with the possibility that oral and gastrointestinal microbiota might be involved in inflammatory processes responsible for atherosclerosis. OTUs attributed to Fusobacterium from the oral cavity correlated with total serum cholesterol and LDL, and Streptococcus OTUs correlated with HDL levels. However, there was no clear fecal microbial signature that could distinguish between patients and controls [40]. In contrast, Karlsson et al showed that individuals with symptomatic atherosclerosis had higher abundance of the genus Collinsella and lower abundance of Eubacterium and Roseburia compared with healthy controls. Functionally, the gut microbiome of patients had increased capacity for peptidoglycan synthesis, which might contribute to the chronic inflammation in the atherosclerotic arterial walls [41]. Another study found an association between coronary artery disease (CAD) and gut microbiota composition by demonstrating that the phylum Bacteroidetes was decreased and the order Lactobacillales was increased in CAD patients compared to both subjects with coronary risk factors but without CAD and healthy controls [42].

Several studies have shown that the fecal microbiota composition in subjects with NAFLD differs from that of healthy controls and obese patients without fatty liver disease [43–45]. Hoyles et al recently show that patients with steatosis have decreased microbial gene richness and altered genetic potential for several functions including the processing of dietary lipids [46]. Furthermore, changes in fecal microbiota composition have been shown to associate with the severity of NAFLD and its progression to fibrosis and non-alcoholic steatohepatitis (NASH) [47, 48]. An increased abundance of *Bacteroidetes* in patients with fibrosis or NASH is the most consistent finding in these studies.

## Mechanisms linking gut microbiota and host lipid metabolism

### Short-chain fatty acids

**Fig. 2:**
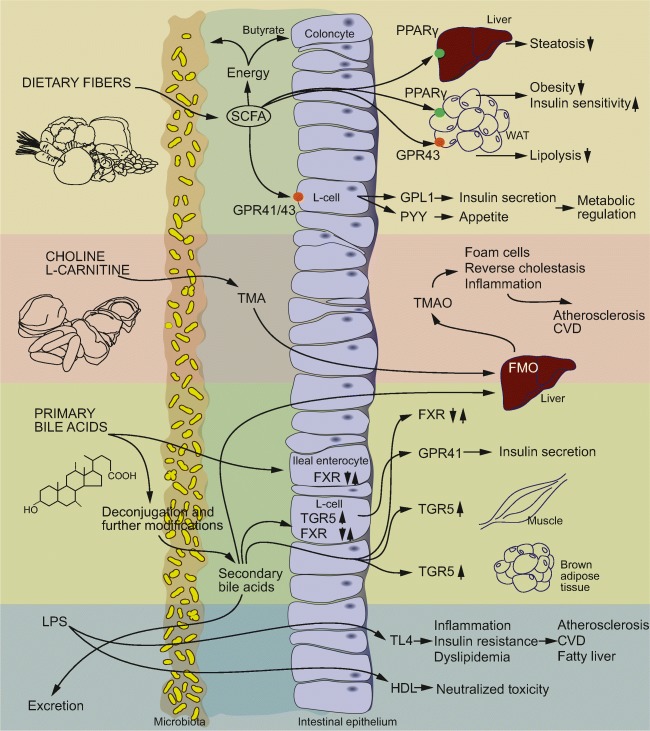
**Mechanisms linking the gut microbiota to lipid metabolism and pathophysiological conditions associated with dyslipidemia.** Short-chain fatty acids regulate host lipid metabolism by supplying the host with energy, improving peripheral tissue metabolism and stimulating incretin hormone production. The gut microbiota transforms choline and L-carnitine to trimethylamine (TMA). TMA is transformed into trimethylamine N-oxide (TMAO) that may promote increased atherosclerosis through mechanisms related to lipid metabolism and inflammation. Bile acids regulate metabolism by binding to farnesoid X receptor (FXR) and G protein-coupled bile acid receptor 1 (TGR5) in several different tissues. Deconjugation of bile acids reduces absorption and increase excretion of bile acids. Increased gut permeability facilitates translocation of lipopolysaccharide (LPS) over the intestinal epithelium. LPS induce inflammation through TLR4 that may result in metabolic perturbations and contribute to development of metabolic diseases. HDL may neutralize the toxic effect of LPS. PPARγ, peroxisome proliferator-activated receptor gamma; GPR43, G-protein coupled receptors GPR43(FFAR2); GPR41, G-protein coupled receptors GPR43(FFAR3); SCFA, short-chain fatty acid; PYY, peptide YY; TLR4, toll-like receptor 4

Short-chain fatty acids (SCFAs) such as acetate, propionate and butyrate are bacterial metabolites derived from fermentation of fibers in the colon (Fig. 2). Both butyrate and propionate have low systemic concentrations whereas acetate levels are higher [49]. SCFAs are important for host metabolism and are used as substrates for energy production, lipogenesis, gluconeogenesis and cholesterol synthesis [50, 51]. Butyrate is an energy source for colonocytes while propionate is mainly metabolized by the liver.

In addition to being metabolic substrates, SCFAs act as signaling molecules, notably through the G-protein coupled receptors GPR43/FFAR2 and GPR41/FFAR3. GPR43 protects against diet-induced-obesity in mice [52–55]. Activation of GPR43 on L-cells increases secretion of glucagon-like peptide-1 (GLP-1) [52, 56] and acetate induces anti-lipolytic activity [57] and improves glucose and lipid metabolism [53] through GPR43 in WAT. GRP41 has also been shown to regulate metabolism through interaction with the gut microbiota. CONV-R *Gpr41* knockout mice are leaner and weigh less than their wild-type littermates, while these differences are not found in GF mice. Furthermore, the microbiota increases peptide YY (PYY) production through GPR41 [58]. Butyrate and propionate have also been shown to activate PPARγ [59], and SCFA-induced activation of PPARγ modulates lipid metabolism through increased energy expenditure [60], reduced body weight and decreased liver triglyceride accumulation [61].

Overall, SCFAs have been shown to have a positive impact on metabolic health [62]. Supplementation with acetate reduces weight gain and improves glucose tolerance in obese and diabetic rats [63], butyrate protects against obesity and increases thermogenesis in mice [60] and propionate or butyrate improves glucose homeostasis in mice [64]. Some minor clinical trials have also found beneficial effects of SCFA or fiber supplementation on body weight [56, 65]. Ingestion of a propionate precursor increases postprandial plasma PYY and GLP-1 and reduces energy intake while long-term treatment results in a reduction in weight gain [56]. Plasma concentrations of PYY and GLP-1 are also increased by acetate in humans [66].

### Bile acids

Primary bile acids are synthesized from cholesterol and conjugated to taurine or glycine in the liver. The bile acids are stored in the gallbladder and excreted into the duodenum after food ingestion to aid emulsification of dietary lipids. Most of the bile acids are reabsorbed and recirculated to the liver, but bacterially mediated deconjugation of the glycine or taurine group reduces reabsorption. Deconjugated bile acids can be further metabolized to secondary bile acids through dehydrogenation, dehydroxylation and epimerization by colonic bacteria [67]. Microbial processing results in a more hydrophobic bile acid pool and facilitates excretion in the feces. Fecal excretion of bile acids is a major sink for cholesterol and bile acids lost in the process need to be replaced by *de novo* synthesis from cholesterol [67].

In addition to their role in lipid digestion, bile acids can act as signaling molecules that regulate host metabolism by binding to the nuclear receptor farnesoid X receptor (FXR) and the Takeda G-protein coupled bile acid receptor TGR5. Microbial processing of bile acids increases the diversity of the bile acid pool and the different bile acids vary in their affinity to the receptors and can act as agonists or antagonists. Both of the primary bile acids cholic acid (CA) and chenodeoxycholic acid (CDCA) and the secondary bile acids lithocholic acid (LCA) and deoxycholic acid (DCA) are FXR agonists, but with different affinities [68]. In humans, CDCA can be transformed into ursodeoxycholic acid (UDCA), which is a FXR antagonist [69]. Furthermore, the taurine-conjugated murine bile acid TβMCA, but not its deconjugated counterpart βMCA, is a potent FXR antagonist [70].

FXR is involved in the regulation of lipid metabolism, especially triglyceride trafficking, synthesis and utilization [71]. Microbial processing of bile acids may therefore influence lipid metabolism through interaction with FXR. By feeding wild-type and *Fxr* knockout mice with or without bacteria a high-fat diet, Parséus et al showed that microbiota-induced weight gain, steatosis and inflammation were dependent on FXR signaling [72]. FXR also changed the gut microbiota composition, and transplantation of the gut microbiota into GF mice transferred the lean phenotype of *Fxr* knockout donor mice, demonstrating that FXR may contribute to increased adiposity by altering the microbiota composition. Comparison of whole body and tissue-specific *Fxr* knockout mice have revealed that activation of the liver and intestinal FXR result in distinct metabolic outcomes in obesity models [73–77]. Several studies indicate that inhibition of intestinal FXR improves metabolic phenotypes [75, 77] but the underlying mechanisms are still unknown.

Bile acids have also been shown to influence host lipid metabolism through TGR5. TGR5 activation in skeletal muscle and brown adipose tissue promotes energy expenditure [78]. In addition, TGR5 signaling induces GLP-1 release from enteroendocrine L-cells, resulting in improved liver and pancreatic function in obese mice [79], with potential influences on lipid synthesis and storage. The microbially produced bile acids LCA and DCA act as agonists to TGR5 [78, 80] but the impact of the gut microbiota on host metabolism via TGR5 remains to be determined.

Bile acids have been implicated in the pathogenesis of fatty liver disease. Patients with NASH have been shown to have altered fecal bile acid composition [81]. In addition, an inverse relation between fibroblast growth factor 19 (FGF19), an FXR-regulated hormone produced in the ileum, and NASH has been reported [82, 83] . The importance of understanding the interplay between the gut microbiota, bile acids and lipid homeostasis is highlighted by efforts to use bile acids as treatments for NAFLD and NASH. One example is UDCA, which has been shown to have beneficial impact on steatosis and serum lipid levels after short-term treatment in severely obese patients [69] while others have reported negative results regarding improvement of NASH in response to UDCA treatment [84, 85]. Another recent example is the semi-synthetic FXR agonist obeticholic acid, which has been shown to improve NASH after 72 weeks’ treatment in a randomized, controlled clinical trial [86]. However, accompanying elevations in serum LDL levels have raised the question of the overall benefit of such treatment. Short-term treatment with the FGF19 analog NGM282 resulted in reduction of steatosis in NASH patients [87]. To date, no clinical study targeting the gut microbiota to specifically modify FXR signaling in NAFLD or NASH has been performed.

### Lipopolysaccharides, gut permeability and inflammation

Lipopolysaccharides (LPS), also known as endotoxins, are structural compounds in the outer membrane of Gram-negative bacteria. LPS induces inflammation through activation of TLR4, which is expressed on immune cells such as macrophages as well as on many other cell types including hepatocytes and adipocytes. The intestinal epithelium works as a barrier to prevent translocation of bacterially derived factors. However, weight gain, high-fat diet [88] and increased exposure of fatty acids [89, 90] may disrupt the gut barrier function allowing translocation of LPS [91, 92]. This results in moderately increased levels of LPS in the blood which is defined as metabolic endotoxemia [91], a condition linked to metabolic perturbations such as dyslipidemia, insulin resistance, NAFLD and cardiovascular disease [93].

LPS interacts with blood lipids in various ways. First, it increases the concentration of blood triglycerides by multiple mechanisms. In rats, low-dose LPS increases hepatic synthesis of VLDL, whereas high-dose LPS decreases lipoprotein catabolism [94]. Mice lacking the TLR4 co-receptor CD14 are resistant to hyperinsulinemia, insulin resistance and steatosis induced by a high-fat diet or LPS [91]. Second, plasma lipoproteins, in particular HDL, have the ability to neutralize the toxic effects of LPS [95, 96]. The capacity of HDL to bind LPS may protect against inflammation. This is supported by the observations that infusion of HDL prior to a LPS challenge reduced release of proinflammatory cytokines in humans [97] and that LPS induces higher levels of TNFα in hypolipidemic rats compared with controls [98].

LPS has been shown to promote atherosclerosis and cardiovascular disease. LPS-treated hypercholesterolemic rabbits have increased atherosclerosis compared with controls [99] and mice lacking TLR4 have reduced atherosclerosis and plaques with decreased amounts of lipid [100]. In humans, high LPS levels during chronic infections are predictors of increased atherosclerotic risk [101] and metabolic endotoxemia increases the risk for cardiovascular disease and mortality in patients with chronic kidney disease [102]. Human TLR4 mutations have been shown to be associated with a decreased response to LPS [103], reduced risk of carotid artery atherosclerosis [104] and acute coronary events [105].

Mouse studies have shown that hepatic steatosis is induced by a high-fat diet and associated with dysbiosis and increased intestinal permeability [91]. Moreover, chemically induced colitis in rats increases the levels of circulating LPS and worsens steatohepatitis during high-fat diet[106]. Dysbiosis-induced permeability increases the levels of TLR ligands in the portal vein, thereby activating hepatic Kupffer cells and stellate cells to stimulate pro-inflammatory and pro-fibrotic pathways via inflammatory cytokines[107, 108]. In addition, mucosal TLR activation appears to contribute to hepatic steatosis via the TLR adaptor MYD88 expressed in the intestine[109]. Mice with an intestinal epithelial cell-specific deletion of MYD88 fed a high-fat diet have improved glucose homeostasis and decreased hepatic lipid content compared with wild-type mice[110]. TLR signaling in the mucosa can also induce production of inflammasomes, multiprotein oligomers responsible for the activation of inflammatory responses. Inflammasomes activate a variety of pro-inflammatory and pro-fibrotic processes involved in the progression of liver disease[111]. For example, activation of the NLRP3-inflammasome by LPS via TLR4 and TLR9 is involved in the development of fibrosis in NAFLD[112]. Gut permeability has also been linked to NAFLD in humans. NAFLD patients have been shown to have increased gut permeability compared to healthy controls and gut permeability correlated with severity of steatosis but not with steatohepatitis in patients with NAFLD[113]. Interestingly, patients with steatosis have also been shown to have a gut microbiota with increased genetic potential for biosynthesis of endotoxin[46].

### Trimethylamine/Trimethylamine N-oxide

The gut microbiota metabolizes methylamine-containing nutrients such as choline, lecithin and L-carnitine to generate trimethylamine (TMA), which is further processed to trimethylamine N-oxide (TMAO) by flavin monooxygenases (FMO) in the liver. TMAO levels have been correlated with risk of cardiovascular events[114] and prevalence of cardiovascular disease[115, 116]. Plasma TMAO levels in different mouse strains have been positively correlated with lesion size [117] and transfer of microbiota from high- and low-TMAO-producing mice to atherosclerosis-prone *Apoe* knockout mice show that increased microbial capacity for TMA production increases aortic lesions[118].

FMO3 is the primary enzyme converting TMA into TMAO. Knockdown of *Fmo3* results in reduced atherosclerotic lesion areas, altered lipid and cholesterol metabolism, and decreased TMAO plasma levels [119, 120]. FMO3 expression is regulated by bile acids by a mechanism that involves FXR [121]. Gut microbiota processing of bile acids could therefore be an alternative mechanism by which the gut microbiota regulates TMAO production.

The mechanisms by which TMAO contributes to atherosclerosis appears to be complex and not fully understood. Antibiotic treatment reduces production of TMA and has been shown to suppress foam cell formation. TMAO can also contribute to atherosclerosis by inhibiting reverse cholesterol transport [115] and by inducing atherosclerosis-promoting inflammatory proteins in vascular cells[122]. In addition to atherosclerosis, increased levels of TMAO are also associated with a frequency of thrombotic events and platelet activation[123].

Although many studies have reported associations between plasma levels of choline, TMAO, and cardiovascular disease [114, 116, 124, 125] the role of TMAO in disease development is still under debate. A recent study comparing conventional and GF *Apoe* knockout mice fed diets with or without choline supplementation found no effect of choline enrichment on aortic root atherosclerosis in mice[126]. TMAO production was dependent on the presence of intestinal bacteria but no relationship between TMAO levels and lesions was observed. These results are in contrast to Wang et al who found a significant correlation between TMAO and lesion size[116]. Similarly, studies using diets supplemented with L-carnitine, which result in increased levels of TMAO, have shown opposite effects on aortic lesions in two different laboratories, further emphasizing context dependency on experimental outcome[115, 127].

## Clinical interventions for treatment of dyslipidemia and NAFLD targeting the gut microbiota

The gut microbiota has been targeted for treatment of diseases related to dyslipidemia. Strategies include supplementing the diet with fibers to enhance the growth or activity of beneficial bacteria (prebiotics), live bacteria (probiotics) or a combination of pre- and probiotics (symbiotics).

A meta-analysis of 11 minor clinical trials using fermented milk and probiotics show beneficial effects on serum lipid profiles[128]. Another meta-analysis focusing on studies using Lactobacillus formulations found an improvement of total serum cholesterol and LDL, but not triglycerides or HDL[129]. One study found improvement of triglycerides but not cholesterol after short-term co-administration of Bifidobacteriae and Lactobacilli strains in healthy subjects, whereas another study observed a decrease in total cholesterol, LDL and triglycerides as well as an increase in HDL using a Bifidobacterium/yeast extract symbiotic[130]. A recent meta-analysis of studies on treatment of NAFLD targeting the gut microbiota showed an overall reduction of AST and ALT levels when pooling data from 25 clinical trials using pre-, pro or symbiotics [131].

Statins lower cholesterol levels by inhibiting HMG-CoA reductase, but may also exert a lipid-lowering effect through interaction with the gut microbiota. Liu et al demonstrated that the cholesterol-lowering effect of rosuvastatin was reflected in microbial alpha diversity measured after eight weeks of treatment [132]. Studies in mice have also shown that statins affect the gut microbiota [133, 134].

The studies performed on microbiota-targeted therapy against dyslipidemia and NAFLD are heterogeneous, the cohorts small and the intervention periods short. Therefore, long-term benefits remain uncertain. Prevention of atherosclerosis by modulation of the gut microbiota has not been studied in humans and data in mice are conflicting[135–137].

## Conclusion and future perspectives

An intricate crosstalk links the gut microbiota, dietary lipids and host lipid metabolism. The microbiota processes lipids and other nutrient factors to produce metabolites with impacts on host lipid homeostasis and putative effects on pathophysiological processes. Studies in gnotobiotic and genetic mouse models have identified mechanisms behind these interactions, and studies in humans have found associations between microbial composition, lipid profiles and prevalence of metabolic diseases. However, although it is evident that fat from different sources has different effects on the gut microbiota, the role of specific fatty acids is not known. It also remains to be investigated how the combination of lipids with other nutrients - such as dietary fibers – affects the gut microbiota. Even though efforts have been made to understand how dietary pattern affect the gut microbiota[138, 139], the importance of specific foods and combinations of nutrients in shaping microbial profile remains elusive. The association between diet, gut microbiota structure and dyslipidemia needs to be studied in large human cohorts to develop therapeutic strategies. Given the individual differences in gut microbiota composition, it is likely that these strategies will require patient stratification and individual-based therapies.
